# Differential Urinary Proteomic Analysis of High-Risk Cervical Intraepithelial Neoplasia

**DOI:** 10.3390/ijms24032531

**Published:** 2023-01-28

**Authors:** Peter Bober, Soňa Tkáčiková, Ivan Talian, Peter Urdzík, Silvia Toporcerová, Ján Sabo

**Affiliations:** 1 Department of Medical and Clinical Biophysics, Faculty of Medicine, University of Pavol Jozef Šafárik in Košice, Trieda SNP 1, 04011 Košice, Slovakia; 2 Department of Gynaecology and Obstetrics, Faculty of Medicine, University of Pavol Jozef Šafárik in Košice, Trieda SNP 1, 04011 Košice, Slovakia

**Keywords:** urine, cervical intraepithelial neoplasia, extracellular matrix, heparan sulfate proteoglycans, proteomic analysis

## Abstract

Human papillomavirus (HPV)-associated lesions and malignancies exhibit alterations in the composition and functionality of the extracellular matrix (ECM) that represent the complex molecular pathways present between infection and disease. A total of 20 urine samples were used, including from 10 patients with cervical intraepithelial neoplasia grade 3 (CIN3) and 10 healthy controls to perform the label-free quantitative analysis using the nano-HPLC and ESI-MS ion trap mass analyzer and matrix-assisted laser desorption ionization–time-of-flight mass spectrometry (MALDI-TOF/MS) fast screening. Among 476 identified/quantified proteins, 48 were significantly changed (log_2_-fold change ≥1.0 or ≤−1.0, −log10 (bbinominal, *p*-value ≥ 1.3), of which were 40 proteins (down-regulated) and 8 proteins (up-regulated) in CIN3, in comparison to healthy controls. The biological function and key pathway enrichment of the gene set using gen set enrichment analysis (GSEA) were analyzed. The ECM-receptor interaction pathway (NES = −1.64, *p* = 0.026) was down-regulated by 13 proteins (HSPG2, COL6A1, COL6A3, SPP1, THBS1, TNC, DAG1, FN1, COMP, GP6, VTN, SDC1, and CD44; log_2_ FC range from −0.03 to −1.48) for the CIN3 group in the KEGG database. The MALDI-TOF/MS screening showed the difference of protein profiles between the control and CIN3 groups, i.e., using the scatter plot with a well-separated shape, as well as effectively distinguishing both groups (control and CIN3) using genetic algorithms (GA) with cross-validation (51.56%) and recognition capability (95.0%). Decreased levels of ECM-receptor interaction proteins may cause disturbances in the interactions of cells with the ECM and play an important role in the development and progression of cervical cancer.

## 1. Introduction

Cervical cancer is most commonly caused by HPV, a tiny, non-enveloped dsDNA virus belonging to the Papillomaviridae family [1]. In terms of cancer-related fatalities among women globally, cervical cancer ranks fourth [2]. More than 90% of high-grade squamous intraepithelial lesions (HSIL or CIN2+) include HPV, which is found in nearly all cervical cancer biopsy samples [3]. Currently, more than 200 different HPV genotypes are known [4]. Of them, HPV 16 and HPV 18 constitute the high-risk oncogenic genotypes, since they are responsible for over 70% of all cervical cancer [5,6,7].

Cervical intraepithelial neoplasia (CIN) presents a precursor lesion where the cervical cancer development begins. High-grade precancerous lesions (CIN2 and CIN3) may appear 3 to 5 years after persistent, high-risk HPV infections. The precancerous lesion of a more advanced cervical cancer, CIN3 (carcinoma in situ-CIS), is a morphologically heterogeneous disease [8].

The dysregulation of proteins that alter the ECM and abnormalities in cell–ECM interactions are key factors in the initiation and progression of many kinds of cancers [9]. Malignant transformation is thought to be characterized by a changed interaction between the ECM and tumor cells [10]. In HPV-associated lesions and malignancies such as cervical cancer, structural and functional alterations of the ECM are frequent [8].

The effectiveness of urine-based, high-risk human papillomavirus (hrHPV) testing for CIN3 identification is less obvious in contrast to the cytology or histology evaluations of cervicovaginal samples, since studies employ a variety of hrHPV assays and non-standardized urine collection methods [11,12]. Nevertheless, due to its low cost, non-invasive nature, and ease of collection, urine samples may be utilized for women who choose not to undergo regular tests [13,14].

In order to compare the protein expression profiles between the urine of healthy controls and CIN3 patients, label-free quantification using nano-HPLC coupled with ion trap mass analyzer and the MALDI-TOF/MS technique was applied in the present study. Currently, literature on scientific studies clarifying the connection between HPV infection and CIN3 status and comparing the proteomic profiles of CIN3 patients to the healthy controls in urine samples are limited. Consequently, our study may assist in elucidating possible biomarkers of CIN3 from urine samples.

## 2. Results

### 2.1. Urine Proteomic Analysis Using the MALDI-TOF/MS and ESI-MS Ion Trap

The total of 20 urine samples, including 10 patients with CIN3 and 10 healthy controls, were used. The difference in the protein profiles between the control and CIN3 groups obtained by MALDI-TOF/MS fast screening analysis was recorded. Proteomic analysis was carried out using the label-free quantitative approach (spectral count) by nano-HPLC and ESI-MS ion trap mass spectrometer.

### 2.2. MALDI-TOF/MS Detection Model Generation

Classification models were generated using three algorithms (GA, SNN and QC) to distinguish the control group and the CIN3 group in the urine samples. For all these models, to classify mass spectra, the default setting was used. Specifically, for the GA model: maximal number of peaks in the model—10, maximal number of generations—50, number of neighbors—3, initial number of peak combinations—automatic detection, mutation rate—0.2, and crossover rate—0.5; for SNN: upper limit of cycles—10 × 100 and automatic detection of prototype number; and for QC: *p*-value *T*-Test/ANOVA.

Cross-validation (CV) and recognition capability (RC), which represent each model’s performance, were employed. In MALDI TOF/MS analysis, the model with the highest RC value was used. The GA model had the highest value of recognition capability (95.0%). In contrast, the model with the highest cross-validation value (62.66%) was the QC model. However, SNN and GA classification models had very comparable cross-validation values, which were 51.02% and 51.56%, respectively (Table 1).

**Figure 1:**
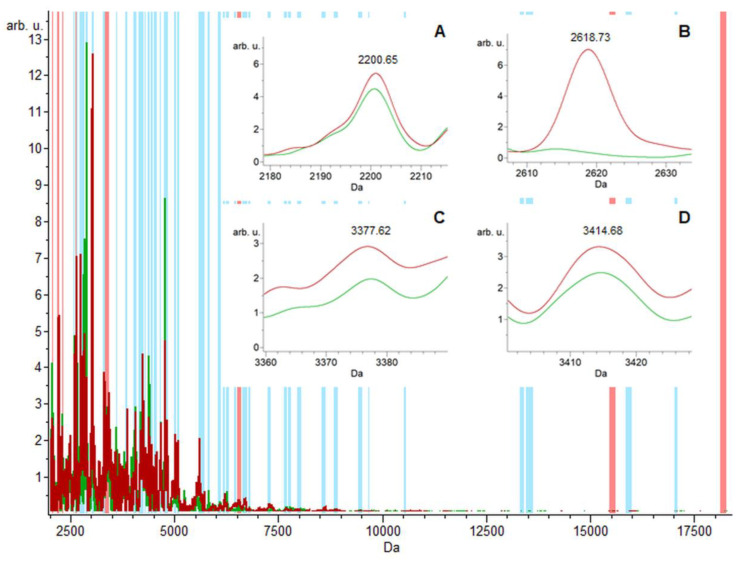
The location of discriminating peak masses, *m*/*z* 2050.8, 2200.6, 2291.0, 2618.7, 3343.6, 3377.6, 3414.6, 6525.5, 15,514.5, and 18,202.8, determined by the GA model with the highest recognition capability. (**A**–**D**) The zoomed-in total average spectra of several ions displaying differential expression levels in the groups; control and CIN3, depicted in red and green, respectively, of the urine samples.

The 10 mass peaks/integration areas with the highest recognition capability were chosen using the GA classification model and are depicted by red bars (*m*/*z* 2050.8, 2200.6, 2291.0, 2618.7, 3343.6, 3377.6, 3414.6, 6525.5, 15,514.5 and 18,202.8) in the urine samples (from a total of 60 mass peaks indicated by blue bars) (Figure 1). Differences between the control and the CIN3 groups in the expression levels of the total average spectra of several ions (in red and green, respectively) generated by the GA model are depicted in Figure 1A–D as zoomed-in small graphs.

### 2.3. MALDI-TOF/MS Cluster Analysis

**Figure 2:**
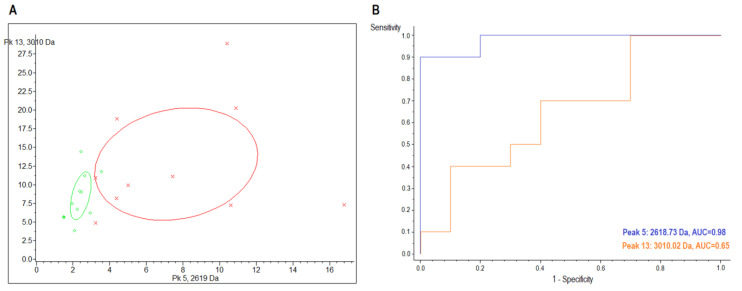
(**A**) Scatter plot of the urine samples determined by the combination of peptide/protein, with *m*/*z* 2618.7 (x-axis) and 3010.0 (y-axis) (peaks with lowest *p*-values) between healthy control subjects (red) and CIN3 (green) patients. Confidence intervals of 95% are shown by ellipses. (**B**) ROC curve plots showing the correlation between the sensitivity and specificity of the *m*/*z* 2618.7 (blue) and 3010.0 (orange). Areas under the ROC curves (AUC) are 0.98 and 0.65, respectively.

The cluster analysis by 2D peak distribution of the peaks at 2618.7 and 3010.0 *m*/*z* manifested their discriminating capability between the CIN3 group and the control group in the urine samples (Figure 2A, Table 2).

### 2.4. ESI-MS Analysis and Label-Free Quantitative Comparisons of Differentially Expressed Proteins in the Urine between CIN3 and Healthy Controls

**Figure 3:**
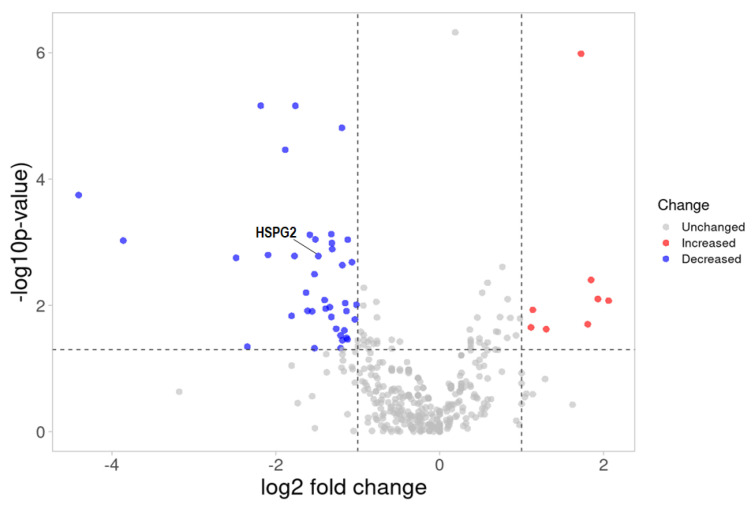
Volcano plot of the 476 differentially expressed proteins between the CIN3 and healthy controls. The 48 proteins that were significantly changed (*p*-value less than 0.05 and log_2_-fold change ≥1.0 or ≤−1.0), i.e., 8 up-regulated (red) and 40 down-regulated (blue) proteins. Volcano plots were generated with the VolcaNoseR online web tool with R/Shiny (https://huygens.science.uva.nl/VolcaNoseR, accessed on 16 November 2022).

We performed label-free quantitative proteomic analysis to identify potential biomarkers in urine samples. Among the 476 total proteins identified/quantified, 48 proteins were significantly altered (log_2_-fold change ≥1.0 or ≤−1.0, −log10 (bbinominal, *p*-value ≥ 1.3), of which there were 40 proteins that were down-regulated and 8 proteins that were up-regulated in the CIN3 group, in comparison to the healthy controls (Table S1 (see Supplementary Materials), Figure 3).

### 2.5. ESI–MS Analysis and Pathway Enrichment Analysis of the Differential Urine Proteins in CIN3 versus Control Samples

**Figure 4:**
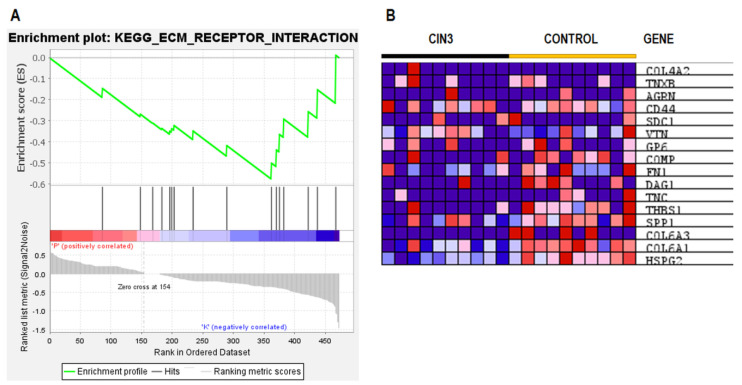
GSEA analysis of the whole, quantified proteins between the CIN3 patients and the healthy control groups. (**A**) Enrichment plot: KEGG ECM-receptor interaction (NES = −1.64, *p* = 0.026). (**B**) Heatmap of core enrichment genes in the gene set of the ECM-receptor interaction. Expression values are represented as colors, where the range of colors (red, pink, light blue, and dark blue) represent the range of expression values (high, moderate, low, and lowest).

**Figure 5:**
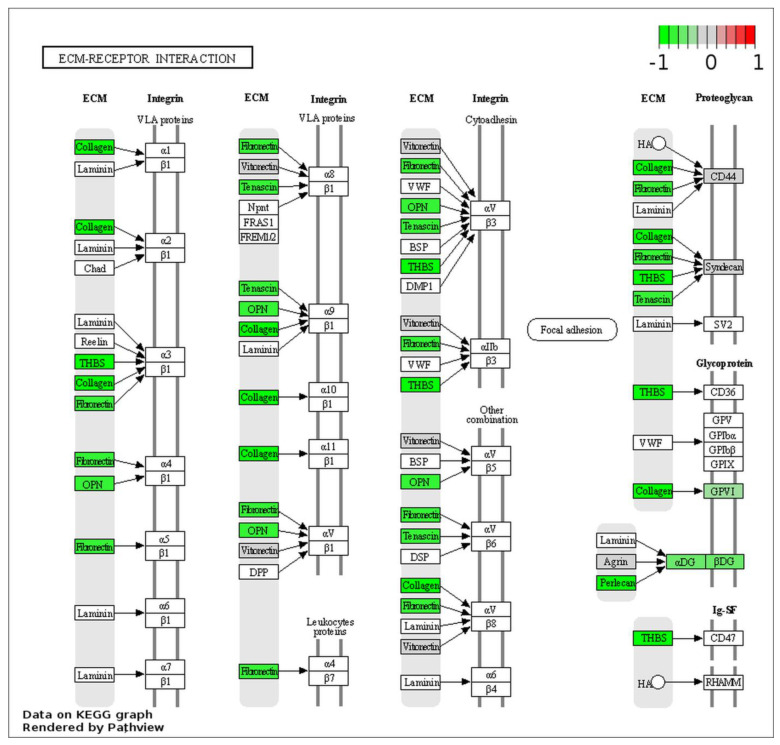
Regulation of genes involved in ECM-receptor interaction (KEGG pathway: 04512). Colored boxes represent differentially expressed genes found in this pathway. The colorbar indicates scaled, log_2_-fold change values (red for upregulated, green for downregulated) in the gene expression of CIN3, in comparison to the control group.

The biological function and key pathway enrichment of the gene set between control and CIN3 in the urine samples were analyzed using GSEA in the KEGG database. As a result, the enriched pathway was obtained, namely the ECM-receptor interaction (normalized enrichment score; NES = −1.64, *p* = 0.026) shown on Figure 4A. The 16 core enrichment genes of the ECM-receptor interaction are shown in Figure 4B. In this pathway, a remarkably high number of genes were altered, e.g., of the all 88 proteins in the ECM-receptor interaction pathway, 15 showed a regulatory change using the Pathview Web Server (Figure 5). Among them were seven core enrichment proteins, i.e., a subset of genes that contributed most to the enrichment result (HSPG2, COL6A1, COL6A3, SPP1, THBS1, TNC, and DAG1). We found that the ECM-receptor interaction pathway was downregulated by 13 proteins (HSPG2, COL6A1, COL6A3, SPP1, THBS1, TNC, DAG1, FN1, COMP, GP6, VTN, SDC1, and CD44; log_2_ FC range from −0.03 to −1.48) and up regulated by 2 proteins (TNXB and COL4A2; log_2_ FC = 0.09 ─ 0.14) for the CIN3 group in the KEGG database. Among them, only HSPG2 was significantly changed (log_2_ FC = −1.48, *p*-value = 0.002) (Table S1, Figure 4B).

## 3. Discussion

Using a wide range of hrHPV DNA tests, several studies examined the hrHPV concordance between urine vs vaginal self-samples and cervical specimens collected by the doctor [15,16,17,18,19,20,21]. However, the direct comparison of different hrHPV DNA tests within the same research has received minimal attention [18,21]; hence, no relevant conclusions about their effects exists. MS-based proteomics has recently become a more potent technique for protein quantification, as well as their identification [22]. By comparing protein expressions across different types of samples or treatments, protein quantification can boost the quantity of relevant information [23]. 

Thanks to MALDI-TOF/MS analysis, the differences in protein profiles between the control and CIN3 groups were confirmed, and both groups (control vs. CIN3) were effectively distinguished. In most cases, the highest mass peak intensity (total average spectra) in the control group, compared to CIN3, were detected using the GA model with the highest sensitivity (95.00%) (Table 1, Figure 1). Moreover, MALDI-TOF/MS showed a potential to be used as a fast and simple screening method for CIN3 identification in urine samples.

Our ESI–MS results of the label-free quantification in the compared groups confirmed the hypothesis of the possibility of classifying the samples (control vs. CIN3) using MALDI-TOF/MS based on their different urine proteomic profiles. The results presented in Table S1 and Figure 5 indicate decreased levels of the 13 proteins (HSPG2, COL6A1, COL6A3, SPP1, THBS1, TNC, DAG1, FN1, COMP, GP6, VTN, SDC1, and CD44) in the ECM-receptor interaction signaling pathway (NES = −1.64, *p* = 0.026) (Figure 4). However, only one protein (HSPG2) with significant change (log_2_ FC = −1.48, *p*-value = 0.002) was detected (Figure 3). From the ECM-receptor interaction pathway analysis, it can be concluded that a decrease in the levels of ECM proteins may cause disturbances in the interactions of cells with the ECM and plays an important role in the development and progression of cervical cancer.

For healthy growth and tissue homeostasis, interactions between cells and the surrounding matrix, as well as within the ECM, are essential [24]. ECM interactions are complex, generating signals that control key cellular activities such as adhesion, differentiation, motility, autophagy, proliferation, and apoptosis [25,26,27]. These cellular processes are regulated by the ECM-receptor interaction pathway [28]. The progression of several malignancies, including breast cancer, prostate cancer, and gastric cancer, is contributed by this pathway [29,30,31]. Wu et al. discovered that the ECM-receptor interaction pathway was the key pathway during CC development using datasets from the GEO database [32]. Moreover, Ramirez-Torres et al. discovered that the ECM-receptor interaction pathway was down-regulated (NES = −2.17, *p* < 0.01) in cervical cancer, compared to control [33]. HPV-associated lesions and malignancies exhibit alterations in the ECM’s structure and functionality, leading to intricate molecular pathways that link infection with disease [11]. In the last period, the ECM changes in cervical cancer were investigated, including the expressions and activities of galectins, collagens, proteoglycans, laminins, fibronectins, integrins, proteases, and regulators [8]. 

The HPV infection cycle starts with the initial contact, which is mostly mediated by HSPGs that are expressed on the basal keratinocytes’ cellular surface or on the ECM. Thus, HSPG proteins have an important role in the HPV infection in regard to their initial attachment [34,35]. Perlecan (HSPG2), a multidomain protein present in all basement membranes, represents the most significant proteoglycan. Perlecan is a found, natural component of the basal membrane of skeletal muscle and participates in the interaction with α-dystroglycan [35]. The dystroglycan complex’s α-subunit mediates its interaction with components of ECM [36]. The findings of Sgambato et al. showed that a decrease in the expression and function of the dystroglycan complex was associated with the onset of cervical cancer and that it may be crucial in the early stages of the disease [37]. Additionally, according to Zhang et al., cervical cancer patients had considerably lower levels of HSPG2 expression than healthy individuals [38]. Furthermore, the results of Ramirez-Torres et al. showed the downregulation of collagens (COL6A1 and COL6A3) in cervical cancer, compared to the control [33]. Additionally, it was previously demonstrated that lower expression of syndecan-1 (SDC1) correlates with enhanced tumorigenicity, i.e., as CIN developed into microinvasive carcinoma, the SDC1 expression level was decreased [39,40]. More than 50% of invasive carcinomas and CIN2+ lesions had decreased CD44 expression, which may be related to loss of cellular adhesion [41]. It has been established that HPV oncogenes inhibited the fibronectin (FN1) promoter [42] and reduced the expression of thrombospondin-1 (THBS1) as potent angiogenesis inhibitors in human keratinocytes [43].

## 4. Materials and Methods

### 4.1. Patient’s Selection

Written informed consent was obtained from women in the control group and the biopsy-proven cervical intraepithelial neoplasia (CIN3) group for urine sampling utilized for research purposes (EK/06045/2020). Randomized urine samples were taken from the Department of Gynecology and Obstetrics, Faculty of Medicine, University of Pavol Jozef Šafárik in Košice, in the period from 1 September to 31 December 2020. Of these, 10 control samples were obtained from healthy donors, with a mean age of 36 years (range: 27–46 years), and 10 patients with CIN3, with a mean age of 34 years (range: 30–39 years).

### 4.2. Urine Sampling

Urine (50 mL) was collected in a sterile tube. All samples were cooled at 4 °C and processed within 30 min. Subsequently, urine samples were centrifuged (2500× *g*, 4 °C, 45 min) to remove cell debris. Urine samples, prior to freezing (−80 °C), were supplemented with the Calbiochem EDTA-free Protease Inhibitor Cocktail Set III (Merck Millipore, Burlington, MA, USA), at a dilution of 1/1000–1/2000, and sodium azide (NaN_3_, 0.02% *v*/*v*).

### 4.3. Sample and Matrix Preparation for MALDI-TOF/MS Analysis

Vortexing was performed on the urine samples that had been defrosted. A Microcon-30 kDa Centrifugal Filter Unit (Merck KGaA, Darmstadt, Germany) was activated with 100 µL of 0.1% TFA, followed by centrifugation at 14,000× *g* for 10 min. Then, 400 µL of human urine samples (diluted 1:5 with TFA 0.5%) were loaded and centrifuged at 14,000× *g* for 30 min. The protein eluate (≤30 kDa) was collected, desalted, and concentrated with a C18 ZipTip^®^ (Millipore, Billerica, MA, USA), as described in the specification sheet. All samples were acidified with concentrated trifluoracetic acid (TFA) (Sigma-Aldrich, St. Louis, MO, USA) to a pH ≤ 4. After that, 1 µL of the sample was spotted onto a MALDI MTP 384 target plate polished steel (Bruker Daltonics GmbH, Bremen, Germany). One microliter of the matrix solution, which contained α-cyano-4-hydroxycinnamic acid (HCCA) (Sigma Aldrich, St. Louis, MO, USA), diluted in ACN/H2O (1:1, *v/v)* with 2.5% TFA, was applied to the air-dried sample. The MALDI-TOF/MS was used to measure the mass spectra of all samples in the positive linear mode (Bruker Daltonics GmbH, Bremen, Germany). Acquisition parameters were used as follows: *m/z* range of 1500–20,000; a matrix suppression cutoff at *m*/*z* 1000; two 1000-shot laser pulses; and a protein calibration standard (Bruker Daltonics, Bremen, Germany). The ClinProTools software for MALDI-TOF/MS data analysis (version 3.0; Bruker Daltonics GmbH, Bremen, Germany) was employed. Raw data preprocessing included baseline subtraction using the top hat algorithm at minimum the baseline width (10%) and smoothing with 10 cycles of a Savitzky–Golay (S-G) filter with a width of 5 *m*/*z* and a mass range *m*/*z* of 2000 to 20,000.

### 4.4. Sample Processing for LC-MS/MS Analysis

A Centriprep^®^ Ultrafiltration Centrifugal Filter (10 kDa MWCO, Merck KGaA, Darmstadt, Germany) was used, activated with 10 mL of redistilled water, then centrifuged (20 °C, 3000× *g*, 30 min). An amount of 10 mL of the urine samples was added, followed by centrifugation. Subsequently, 0.005 M NaCl (10 mL) was added and centrifuged. The previous step was repeated once more. The obtained supernatant (4 mL) was added to an Amicon^®^ Ultra-4 Centrifugal Filter Unit (3 kDa MWCO, Merck KGaA, Darmstadt, Germany) and centrifuged (4 °C, 7000× *g*, 30 min). The concentration of proteins was determined using the Bradford method with 4 µL of the sample. An amount of 100 µL of 10 mM DTT in 8 M urea (in 100 mM TRIS/HCl, pH 8) was added to the extracted proteins, mixed in a thermomixer (45 min at 37 °C), and centrifuged (1000× *g*, 10 min at 4 °C). Next, 100 µL of 50 mM IAA in 8 M urea (in 100 mM TRIS/HCl, pH 8) was added, thermomixed (30 min at 37 °C in the dark), and centrifuged. The protein solution was washed using ACN/water (1/1, 500 µL), thermomixed (20 °C, 10 min), and centrifuged (2500× *g*, 10 min, 4 °C). Subsequently, the solution was washed with 0.1 M NH_4_HCO_3_ (500 µL) and centrifuged (3500× *g* for 10 min at 4 °C). The previous step was repeated once more. The 0.1 M NH_4_HCO_3_ (100 µL) containing 0.002 M CaCl_2_ and trypsin (35 µL, overnight digestion) was added to the protein solution. Subsequently, the samples were centrifuged (7000× *g*, 55 min, 4 °C). Peptides were acidified with concentrated formic acid (FA) to a pH ≤ 4.

### 4.5. LC–MS/MS Analysis and Database Search

The nanoliquid HPLC (Ultimate 3000 RSLC Nano, Thermo Fisher Scientific, Germering, Germany), coupled with an amaZon speed ETD ion trap mass spectrometer (Bruker Daltonik, Bremen, Germany) interfaced with CaptiveSpray ion source (Bruker Daltonik, Bremen, Germany), was used to analyze the urine samples. The Acclaim^®^ PepMap 100 C_18_ trap column (100 µm × 2 cm, 5 µm particles, 100 Å) was loaded with a 1 µL sample (1 µg of peptides), which included 1 µg of peptides using the mobile phase (98% H_2_O and 2% ACN with 0.1% FA) at an 8 µL min^−1^ flow rate. The following step involved washing the peptide from the trap column to the Acclaim^®^ PepMap RSLC C_18_ analytical column (75 µm × 15 cm, 3 µm particles, 100 Å) and eluting them to ESI-MS. Gradient elution was as follows: a 96% mobile phase A (98% H_2_O and 2% ACN with 0.1% FA) and 4% mobile phase B (95% ACN and 5% H_2_O with 0.1% FA) ratio was kept for 5 min. The 4% mobile phase B increased to 35% mobile phase B in the next 90 mins of the HPLC run. The 95% mobile phase B was kept from 95 to 105 min for a column wash, followed by column equilibration with 4% mobile phase B for 120 min. A 0.4 µL min^−1^ flow rate was used. MS scan parameters: positive ion mode charges, enhanced resolution scans, ion charge control (ICC) at 400,000, as well as maximum accumulation time (50 ms), and an *m*/*z* range from 300 to 1300 were set. MS/MS scan parameters: Xtreme resolution mode, ICC target at 500,000 with the maximum accumulation time of 100 ms and an isolation width of 2.2 *m*/*z* were set. For protein identification, Mascot engine (version 2.4.0, Matrix Science, London, UK) and Database SwissProt (version 2020_02) were used, and the search parameters were: taxonomy—Homo sapiens (human); enzyme—trypsin; fixed modifications—carbamidomethylation of cysteine (C), variable modification—oxidation of methione (M); allowed missed cleavages—up to 2; peptide charge +2 and +3; and minimum peptide length—3. For protein assessment, a false discovery rate (FDR) threshold of ≤1% was used, with the minimum of two unique peptides.

### 4.6. Protein Identification and Label-Free Quantification

Mascot data files (technical duplicates) were loaded into the Proline software (Proline Studio 2.0.1, http://proline.profiproteomics.fr/, accessed on 30 March 2022). A minimum score of 20 and an identity *p*-value of 0.05 were used to validate PSM (peptide–spectrum matches). A maximum FDR of 1% was used for the protein set. Weighted spectral counts were calculated for each protein according to Abacus [44]. 

### 4.7. Pathway Analysis

Gene set enrichment analysis (GSEA) was performed using GSEA v.4.2.3 (Broad Institute, Cambridge, MA, USA, https://www.gsea-msigdb.org/gsea/, accessed on 16 November 2022). We set the parameters and ran the enrichment tests on the gene set database (ftp.broadinstitute.org://pub/gsea/gene_sets/c2.cp.kegg.v2022.1.Hs.symbols.gmt, accessed on 16 November 2022) for the number of permutations (1000) and the permutation type (phenotype). The data of the signaling pathways from the Kyoto Encyclopedia of Genes and Genomes (KEGG; www.genome.jp/kegg, accessed on 16 November 2022) were analyzed using the Pathview Web server (https://pathview.uncc.edu/, accessed on 16 November 2022).

### 4.8. Statistical Analysis

Statistical methods (Student’s *t*-test or a Wilcoxon test) were used to choose discriminative peaks for the MALDI-TOF/MS data processing. The supervised neural network (SNN), genetic model (GA), and quick classifier (QC) mathematical models for classifying mass spectra were used. The model was built using the 10 peaks of the averaged spectra. The label-free protein quantification analysis between groups (control and CIN3) was used. A beta-binomial test was performed using weighted spectral counts, and a *p*-value computed for each protein set employing the R package BetaBinomial 1.2 was integrated into the Proline Studio software [45]. Using GSEA, the FDR < 0.25 and nominal *p*-value < 0.05 were used to identify significantly enriched pathways.

## 5. Conclusions

The MALDI-TOF/MS results confirmed the differences in protein profiles between the control group and the CIN3 group and can be used as a tool to effectively distinguish between these groups. From the ECM-receptor interaction pathway analysis, it can be concluded that the decrease in the levels of ECM proteins, i.e., HSPG2, COL6A1, COL6A3, SPP1, THBS1, TNC, DAG1, FN1, COMP, GP6, VTN, SDC1, and CD44, may cause disturbances in the interactions of cells with the ECM and play an important role in the development and progression of cervical cancer. However, further research will be required to fully comprehend how proteins impact the dynamic equilibrium of the ECM in related diseases. 

## Figures and Tables

**Table 1:** Results of a cross-validation test and recognition capability test of the GA, SNN, and QC models to distinguish the two groups (control and CIN3) in the urine samples.

Samples	Model	Generated Peaks	Cross-Validation (%)	Recognition Capability (%)
urine	GA	10	51.56	95.00
	SNN	15	51.02	90.00
	QC	3	62.66	85.00

**Table 2:** Statistical analysis of the most discriminating protein or polypeptide ions in the cluster analysis by 2D peak distribution.

Sample	Index Peak	Mass (*m*/*z*)	Dave ^1^	PTTA ^2^	PWKW ^3^	PAD ^4^	Control (avg ^5^ ± SD ^6^)	CIN3 (avg ± SD)	AUC
urine	5	2618.73	5.79	0.289	0.0026	0.000219	8.48 ± 4.94	2.69 ± 0.63	0.98
	13	3010.02	4.71	0.59	0.672	0.00872	14.17 ± 8.36	9.46 ± 3.55	0.65

^1^ Difference between the maximal and minimal average peak area/intensity of all classes. ^2^ *p*-value of *t* test. ^3^ *p* value of Wilcoxon test. ^4^ *p*-value of Anderson–Darling test. ^5^ peak area/intensity average of class. ^6^ Standard deviation of the peak area/intensity average of class.

## Data Availability

The data presented in this study are available upon request from the corresponding author.

## Supplementary Materials

The following supporting information can be downloaded at: https://www.mdpi.com/article/10.3390/ijms24032531/s1.
